# Clinical implications and characterization of *Group A Streptoccoccus* infections in adults with cystic fibrosis

**DOI:** 10.1186/s12890-015-0157-1

**Published:** 2015-12-12

**Authors:** Kate Skolnik, Austin Nguyen, Ranjani Somayaji, Christina S. Thornton, Barbara Waddell, Michael G. Surette, Harvey R. Rabin, Michael D. Parkins

**Affiliations:** Departments of Medicine, The University of Calgary, Calgary, AB Canada; Departments of Microbiology, Immunology and Infectious Diseases, The University of Calgary, 3330 Hospital Drive, NW, Calgary, AB Canada; Department of Medicine, McMaster University, Hamilton, ON Canada; Department of Biochemistry, McMaster University, Hamilton, ON Canada; The Farncombe Family Digestive Health Research Institute, McMaster University, Hamilton, ON Canada

**Keywords:** Cystic fibrosis, *Streptococcus pyogenes*, Group A Streptococci, GAS, Pulmonary exacerbation, Eradication, Emerging pathogen

## Abstract

**Background:**

Persistent airway infection is a hallmark feature of cystic fibrosis (CF). However, increasingly it has been observed that non-classical pathogens may transiently infect CF lower airways. *Streptococcus pyogenes* (Group A Streptococcus; (GAS)) is an uncommon but potentially dangerous cause of community-acquired pneumonia. Our aim was to determine the incidence, natural history, and clinical impact of GAS infections in CF and phenotypically and genotypically characterize the isolates.

**Methods:**

We retrospectively evaluated the Calgary Adult CF Clinic biobank to identify adults with at least one GAS isolate. Patient demographics, medical and pulmonary exacerbation (PEx) histories were evaluated. The primary outcome was PEx occurrence at incident GAS culture. Secondary outcomes evaluated were changes in lung function and PEx frequency following GAS isolation. Isolates were assessed for extra-cellular virulence factor production capacity and ability to produce quorum sensing (AI-2). Isolates were genotyped using pulse-field gel electrophoresis (PFGE).

**Results:**

Fifteen individuals who cultured GAS twenty times were identified. At the time of GAS isolation, 47 % (7/15) of subjects experienced a PEx and half of these (4/7) were severe. Individuals were more likely to have a PEx at the time of the index GAS isolate compared to the preceding visit (RR = 6.0, 95 % CI 0.82–43.0, *p* = 0.08), particularly if GAS was the numerically dominant sputum pathogen (RR = 6.5, 95 % CI 1.00–43.0, *p* = 0.009). There were no changes in PEx frequency or rate of lung function decline following GAS. None of the patients developed chronic airways infection, bacteremia, necrotizing pneumonia or empyema. Susceptibility was universal to common anti-Streptococcal antibiotics and anti-Pseudomonal antibiotics commonly used in CF, with the exception of azithromycin. GAS isolates varied in their production of protease, DNase, and AI-2 but these did not correlate with PEx, and none produced elastase, chrondrotin sulfatase or H_2_0_2_. One patient had prolonged carriage with the same isolate and two patients had isolates with similar PFGE patterns.

**Conclusions:**

GAS was an uncommon lower respiratory pathogen of adults with CF. Identification of GAS in sputum was frequently associated with PEx, particularly when numerically dominant. However, transient GAS infection did not result in chronic infection nor appreciably change long-term disease trajectory.

## Background

Cystic fibrosis (CF) is the most common lethal autosomal recessive disease amongst Caucasians [1]. Typically affected organs include the sinuses, lungs, gastrointestinal system and the male reproductive system. Pulmonary complications (both acute and chronic) are the primary cause of morbidity and mortality in the adult CF population [2, 3]. CF lungs are classically characterized by viscous secretions as well as impaired mucociliary clearance [4, 5]. These factors compromise airway clearance creating an optimal environment for bacterial colonization, inflammation, chronic infection, and eventually, bronchiectasis.

The cultured microbiome of the CF respiratory system is well-characterized and unique from other forms of chronic lung disease. Nevertheless, our understanding of lung microbiology in the CF population continues to evolve. Traditional perceptions focused on chronic colonization with classical CF pathogens such as *Pseudomonas aeruginosa, Staphylococcus aureus, Haemophilus influenzae, and Burkholderia cepacia* complex [6]. Other microorganisms such as *Stenotrophomonas maltophilia, Achromobacter* species, and mycobacteria have been identified as emerging CF pathogens in the last two decades [6, 7]. Whereas traditional pathogens like *P. aeruginosa* and *B. cenocepacia* have clearly been associated with adverse outcomes, the clinical implications are less clear with many emerging pathogens [8–10]. Furthermore, it is apparent that CF airways disease may exist in both stable (classical CF pathogens) and dynamic states (non-classical pathogens whose presence in the CF airways disease is usually only temporary). How these transient infections affect CF outcomes is entirely unknown. Consequently, clinicians are posed with unique management challenges when these atypical microbes are isolated from the lower respiratory tracts of CF patients.

One such organism is Group A Streptococcus *(Streptococcus pyogenes)*, a common human pathogen that is rarely observed from CF lungs. *S. pyogenes* are β-hemolytic Gram positive cocci and facultative anaerobes. Its ability to cause hemolysis on blood agar plates allows it to be distinguished from other more indolent streptococci [11].

Furthermore, GAS has numerous virulence factors that enable it to evade host immune defenses, colonize epithelial surfaces, and cause infection [12, 13].

GAS can cause a variety of different infections with a spectrum of disease severity ranging from mild to invasive and life threatening [14]. While it most often manifests as pharyngeal, soft tissue or skin infections, it can rarely manifest as a respiratory infection. Although GAS only accounts for a small proportion of community acquired pneumonia in the general population, it tends to cause more severe and aggressive pulmonary infections and often manifest as empyema [14]. The prevalence, natural history and clinical effect of GAS in the CF population are unknown. Herein we set out to determine the natural history of GAS and outcomes associated with GAS infection in adults with CF. Furthermore, we sought to characterize GAS to determine if genotypic or phenotypic features were associated with disease.

## Methods

### Population

We retrospectively evaluated the Calgary Adult CF Clinic Biobank (CACFB; a prospectively collected and inventoried repository of every bacterial isolate from every CF sputum sample collected from each clinical encounter since 1978). Patients provide prospective consent for collection and research at enrollment into the clinic. Ethics for the collection and analysis was granted by the Conjoint Health Research Ethics Board (E-23087). Patients who cultured GAS at least once were included. There were no exclusion criteria other than age under 18 years.

### Clinical data collection

The study design was retrospective and encompassed the two years preceding and two years following each GAS isolation. We collected baseline patient demographic information, dynamic spirometry data, exacerbation data, medications at the time of GAS isolate, and the presence of CF and non CF-related co-morbidities. Quantitative microbiology (reported as colony forming unit (CFU)/ml of sputum) was performed on each sample as is standard of care within our institution.

The primary outcome was the occurrence of pulmonary exacerbation (PEx) at the time of index GAS isolate compared to the preceding clinical visit. A PEx was defined based on a retrospective chart evaluation to identify documentation which met Fuchs’ criteria and for which acute antibacterial therapy was prescribed [15]. A severe PEx was one requiring intravenous antibiotics and/or hospitalization. Secondary outcomes included: (1) change in predicted forced expiratory volume in one second (FEV_1_) at the time of GAS identification versus the last clinic visit; (2) PEx frequency in individuals before and after the first GAS isolate; (3) rate of FEV_1_ decline before and after GAS, (4) severe complications of GAS infection; and (5) rate of progression to chronic infection.

### Characterization of GAS

Genotyping and phenotyping were carried out on viable GAS isolates. GAS were genotyped using pulsed field gel electrophoresis (PFGE) to assess for clonality using the protocol of Sibley et al. [16]. Restriction digestion was performed independently with both SmaI and ApaI. PFGE profiles were compared using BioNumerics Version 7.0 (Applied Maths, Austin TX). Strains that had banding patterns ≥80 % identical were *a priori* considered related, conforming to the Tenover criteria, where isolates with 1 to 3 band differences were still considered related [17]. Dendrograms were generated at 2.0 % position tolerance using the unweighted pair-group method with arithmetic mean (UPGMA) and the Sørensen-Dice similarity coefficient.

Antibiotic susceptibility testing was performed as per Thornton et al. [18]. Briefly, colonies from a 24-hour incubation on Columbia Blood Agar (CBA) were suspended in 0.85 % saline solution to a 0.5 McFarland standard and spread on the Mueller-Hinton Blood Agar (MHBA) plates with a sterile cotton swab. Antibiotic discs were obtained from Oxoid (Nepean, Ontario) and stored at 4 °C until use. Antibiotics used in this investigation are those commonly used in CF and those frequently prescribed for GAS infection. The following antimicrobial discs were stamped on with a disc dispenser or manually placed: penicillin G (P, 10 U), ceftriaxone (CRO, 30 μg), ceftazidime (CAZ, 30 μg), azithromycin (AZM, 15 μg), erythromycin (E, 15 μg), clindamycin (CDA, 2 μg), and levofloxacin (LEV, 15 μg). The “D-test” for inducible clindamycin resistance was performed. Plates were incubated for 20 to 24 h at 37 °C with 5 % carbon dioxide (CO_2_) after which zone diameters were measured and compared to established breakpoints to determine susceptible (S), intermediate (I) and resistant (R) status as per CLSI guidelines [19, 20].

There were six exo-enzyme assays of GAS virulence factors capable of breaking down host products to aid in bacterial survival performed using cultures grown at 37 °C with 5 % CO_2_; each exo-enzyme assay involved the creation of a media with specific materials to test for the virulence factor as per the methods of Grinwis et al. [21]. Production of virulence factors was scored as positive (production of the factor) or negative (no evidence of this particular factor).

Autoinducer-2 (A1- 2) is a universal quorum sensing molecule enabling interspecies signaling and communication. Cell free supernatants of each isolates were screened using a *Vibrio harveyi* bioassay for detection of the AI-2 using the methods of Grinwis et al. [21, 22]. In brief, overnight cultures of GAS were incubated in Todd-Hewitt-Yeast broth with 0.5 % yeast extract (Bacto) for 24 h at 37 °C with 5 % CO2. 1 ml was transferred to a 1.5 ml tube and centrifuged at 2300 g for 5 min and filtered through 0.2 um membrane. 400 ul was stored at −20 °C until use. The supernatant was mixed with the *Vibrio harveyi* MM32 reporter strain (*luxS*-, *luxN*-, deficient in production of AI-2 but sensitive to its production) and grown for 24 h in Todd-Hewitt- Yeast broth at 30 °C. At 24 h luciferase production (counts per second) was measured using a Wallac Victor2 microplate reader (Perkin Elmer, Waltham, MA) and reported as positive if >2.5 fold-above the negative control. All assays were performed in triplicate and for statistical analysis, Tukey’s multiple comparison test was performed using Prism 5.0.

### Statistical analysis

Demographic data was analyzed using descriptive statistics. Chi-Squared testing was used to determine the relative risk (RR) of PEx at the time of GAS isolation compared to the preceding visit as well as to determine if various clinical or microbiological factors affected the odds of PEx at GAS isolation. Wilcoxon rank- sum tests were used to compare the PEx rates and rate of FEV_1_ change in the 2 years before and 2 years following the index GAS isolates. Nonparametric tests were applied given the small sample size. Analysis was performed with STATA/IC 13.1 software (Stata- Corp, TX, USA).

## Results

### Population characteristics

Between 1978 and 2013, there were fifteen individuals from a cohort of 318 adults with CF (4.7 %) who had GAS isolated from their sputum. Thirteen patients cultured GAS only once, and two patients had repeated cultures for a total of 20 GAS isolates present within the CACFB. One patient (Patient 6) had three consecutive sputum samples growing GAS over 161 days, but subsequently cleared. Another patient (Patient 15) had intermittent GAS in their sputum on four separate occasions spanning 8.6 years. No patient became a chronic GAS carrier.

There were a comparable number of men and women (8 and 7, respectively) with a median age of 26 at the time of first GAS isolate (IQR 19 to 33 years) (Table 1). The majority had baseline airflow obstruction (86 %), with a median FEV_1_ percent predicted of 50 % (IQR 35 to 82.6) (Tables 1 & 2). Most individuals were lifelong-long nonsmokers and had multi-organ CF disease in addition to bronchiectasis (Table 1). At the time of incident culture, half the group (7/15) was on chronic suppressive antibiotics (inhaled tobramycin or oral azithromycin) and a similar number received long-acting inhalers (inhaled corticosteroid, long acting beta agonist, or a combination ICS/LABA) (Table 1). Only one third were using an inhaled mucolytic and/or hypertonic saline (Table 1). Information regarding frequency, quality and nature of chest physiotherapy could not be reliably ascertained.

**Table 1:** Baseline demographics of adult cystic fibrosis patients with known *S. pyogenes* Sputum isolates

Patient	Age^a^	Sex	Mutation 1	Mutation 2	Pancreatic Status	Current Smoker	Baseline FEV^1^ (L)^b^ (% predicted)	Baseline FVC (L)^c^ (% predicted)	TIS^d^	AZM^e^	LABA^f^	ICS^g^	CF^h^ Co-morbidities
1	19	M	F508del	F508del	I	N	2.62 (74)	4.09(100)	Y	N	N	N	DIOS, B
2	19	M	F508del	3849+1G->A	I	N	3.07 (71)	-	N	N	N	N	Liver, S
3	34	M	F508del	E56K	S	N	3.87 (106)	4.98(115)	N	N	N	N	None
4	18	F	M1101K	M1101K	I	N	2.22 (71)	3.06(93)	Y	N	N	N	
5	24	F	F508del	F508del	I	Unknown	1.19 (35)	1.84(47)	N	N	N	N	DIOS
6	28	F	F508del	F508del	I	N	2.39 (89)	3.72(122)	N	N	N	N	IGT,DIOS
7	38	F	F508del	R347H	S	Unknown	2.15 (77)	3.14(95)	N	N	N	N	S
8	48	M	F508del	F508del	I	N	2.30 (54)	4.79(90)	Y	Y	Y	N	IGT
9	19	M	1717-1G->A	Unknown	S	N	3.73 (82)	4.55(85)	N	N	N	N	S
10	21	M	F508del	F508del	I	Y	2.63 (40)	3.61(84)	N	N	N	N	DIOS
11	33	F	F508del	R334W	I	N	1.11 (71)	2.34(74)	N	N	N	N	DIOS
12	26	M	G542x	Unknown	I	Y	2.96 (69)	4.99(100)	N	Y	Y	N	CFRD
13	26	F	Unknown	Unknown	I	N	2.80 (88)	4.10(108)	Y	N	N	N	CFRD,B, S, DIOS,
14	24	M	F508del	F508del	I	N	3.84 (84)	5.80(115)	Y	N	N	Y	Liver
15	28	F	M1101K	M1101K	I	N	2.67 (91)	3.37 (101)	N	N	N	N	B,S

*I* insufficient, *S* sufficient, *Y* yes, *N* no

^a^ Age at time of first *S. pyogenes* isolation.

^b^ *Baseline FEV*_*1*_ forced expiratory volume in one second recorded 2 years prior to the index GAS isolate

^c^ *Baseline FVC* forced volume capacity recorded 2 years prior to the index GAS isolate

^d^ *TIS* Chronic inhaled tobramycin therapy

^e^ *AZM* Chronic azithromycin

^f^ *LABA* Long-acting beta agonist

^g^ *ICS* Inhaled corticosteroid

^h^ *CF* Comorbidities, *DIOS* Distal Ileal Obstruction Syndrome, *B* osteoporosis, *Liver* liver disease, *S* sinus disease, *IGT* Impaired Glucose Tolerance, *CFRD* CF related diabetes

**Table 2:** *S.pyogenes* sputum isolates and exacerbation status in adult cystic fibrosis patients

Isolate	Patient^a^	Date of Isolation	CFU^b^	GAS Most Abundant	Reduced FEV_1_^c^	PEx^d^	Chronic Bacterial Infections
1	1	08-1997	10^6^	N	N	N	PA
2	2	04-2010	10^4^	N	Y	Y^a^	None
3	3	08-2013	10^4^	Y	N/I	Y	None
4	4	09-2010	10^6^	Y	N	N	PA, MSSA
5	5	09-2010	10^7^	Y	N/I	Y	MSSA, HI
6	6	12-2001	10^7^	Y	N	N	MSSA
7	6	02-2002	10^7^	Y	N	N	None
8	6	05-2002	10^7^	Y	N	Y^a^	None
9	7	07-2006	10^7^	Y	Y	Y	MSSA, HI
10	8	07-2012	10^5^	N	N	N	PA
11	9	10-1995	10^8^	Y	N	Y^a^	MSSA, HI
12	10	05-1999	10^7^	N	N	N	MSSA, Bc
13	11	01-2001	10^7^	Y	N	N	MSSA
14	12	10-2012	10^5^	Y	N	N	PA
15	13	11-2012	10^6^	Y	N	Y	PA
16	14	11-2006	10^7^	Y	N	N	PA, MSSA
17	15	10-2000	10^6^	N	N	N	PA, MSSA
18	15	11-2002	10^5^	N	N	Y	None
19	15	02-2009	10^6^	N	N	Y	None
20	15	05-2009	10^3^	N	N	N	None

^a^ Refer to Table 1

^b^ *CFU* colony forming units/ml of sputum

^c^ *Reduced FEV1* reduction in reduction in FEV_1_ by >10% at time of visit compared to baseline FEV_1_

^d^ *PEx* pulmonary exacerbation as defined by Fuch’s Criteria and the need for antibiotics

^*^ *Severe PEx* pulmonary exacerbation requiring hospitalization and/or parenteral antibiotics

^e^ *PA Pseudomonas aeruginosa, MSSA* Methicillin sensitive *S. aureus, HI Haemophilus influenzae, Bc Burkholderia cenocepacia*

*Y* yes, *N* no, *N/I* no information, *N/I* no information

### Primary outcome - risk of pulmonary exacerbation

At the time of first GAS isolation, 47 % (7/15) of individuals experienced a PEx and approximately half of these (4/7) were severe. Of all encounters in which GAS was identified, 45 % (9/20) were associated with a PEx; 44 % (4/9) of which were severe (Table 3). Relative to the preceding clinical visit, patients had a trend towards increased PEx risk at incident GAS isolation (RR = 6.0, 95 % CI 0.82–43.0, *p* = 0.08). If GAS was the numerically dominant organism in the collected sputum, risk was further increased (RR = 6.5, 95 % CI 1.00–43.0, *p* = 0.009); however, there was no specific log threshold for GAS above which PEx was more likely. Risk of PEx was not affected by the individual’s age, sex, severe airflow obstruction at the preceding visit (as defined by % predicted FEV_1_ < 40), or presence of specific chronic cultured microorganisms (as defined by Leeds Criteria) [23]. Furthermore, the risk of PEx was not influenced by the use of chronic antibiotics, inhalers, mucolytics or hypertonic saline (data not shown).

### Exacerbation frequency and lung function

Of the 76 PEx during the study period, 38 occurred before and 29 after the first GAS isolate, respectively. The median number of PEx per patient was 2.5 (IQR 0 to 5) in the two years preceding index GAS isolate, compared to 2 (IQR 0 to 3) in the two following years. GAS did not increase the frequency of PEx in the two years after the index GAS isolate compared to the two preceding years (RR = 1.07, 95 % CI 0.71 to 1.63, *p* = 0.77); the frequency of severe PEx was also unchanged (RR = 1.04, 95 % CI 0.5 to 2.13, *p* = 1.00). In fact, 60 % (9/15) of individuals had a lower PEx frequency after the first GAS isolate.

Only 15 % (2/13) of subjects had a significant decrease in their FEV_1_ (≥10 %) at incident GAS isolate compared to the last visit and a minority of samples collected at any time with GAS (2/17) were associated with a decreased FEV_1_. Furthermore, the rate of FEV_1_ decline did not significantly differ in the two years following the index isolate compared to the two preceding years; this was true for both the decline in absolute FEV_1_, (*p* = 0.27) and % predicted FEV_1_ (*p* = 0.72).

### Clinical outcomes

Bacteremia, necrotizing pneumonia, and empyema were not observed with any of the GAS isolates. In the two years post index isolate, no individuals required initiation of chronic oxygen therapy or listing for lung transplant.

### Phenotypic and genotypic characterization of GAS

Antibiotic susceptibility testing data was available on all isolates to penicillin. Of the 20 isolates available in the biobank, 11 were viable for further phenotypic assessment and were compared against GAS ATCC 19615. All viable GAS strains were sensitive to levofloxacin, penicillin G, and ceftriaxone (Table 3). Azithromycin resistance was rare (8 %), and not observed in patients on chronic macrolide therapy. CLSI breakpoints for GAS do not exist, however ceftazidime (an agent commonly used in CF) demonstrated considerably inferior anti-GAS activity (median KB zone of 33 mm). No clindamycin resistance, or inducible resistance with the D-test was observed. The majority (10/11) of GAS isolates produced DNAse and only one-quarter (3/11) produced elastase (Table 3). None of the GAS isolates produced protease, chondroitin sulfatase, or hydrogen peroxide (Table 3). AI-2 production was observed from all but one GAS isolate. There was no association between the production of particular virulence factors and the occurrence of PEx at isolation (data not shown).

**Table 3:** Antibiotic susceptibility and virulence factors of *S. pyogenes* sputum isolates from adult cystic fibrosis patients

Patient	Iso-late	Antibiotic susceptibility						Virulence Factor Production^b^					
		PEN	LVX	CAZ^a^ (mm)	CRO	CLI	AZM	Protease	Elastase	H_2_O_2_	DNAse	Chondroitin Sulfatase	AI-2
1	1	S	S	38	S	S	S	P	N	N	P	N	N
2	2	S	-	-	-	S	-	-	-	-	-	-	-
3	3	S	-	-	-	-	-	-	-	-	-	-	-
4	4	S	S	37	S	S	S	N	N	N	P	N	P
5	5	S	S	33	S	S	S	P	N	N	P	N	P
6	6	S	-	-	-	-	-	-	-	-	-	-	-
6	7	S	-	-	-	-	-	-	-	-	-	-	-
6	8	S	-	-	-	-	-	-	-	-	-	-	-
7	9	S	S	25	S	S	S	N	N	N	P	N	P
8	10	S	S	30	S	S	R	N	N	N	P	N	P
9	11	S	S	34	S	S	S	N	N	N	P	N	P
10	12	S	S	34	S	S	S	N	N	N	P	N	P
11	13	S	S	40	S	S	S	N	N	N	P	N	P
12	14	S	-	-	-	S	-	-	-	-	-	-	-
13	15	S	S	33	S	S	S	N	N	N	N	N	P
14	16	S	-	-	-	-	-	-	-	-	-	-	-
15	17	S	-	-	-	-	-	-	-	-	-	-	-
15	18	S	-	-	-	-	-	-	-	-	-	-	-
15	19	S	S	35	S	S	S	N	N	N	P	N	P
15	20	S	S	38	S	S	S	N	N	N	P	N	P
ATCC	19615	S	S	31	S	S	S	P	N	N	P	N	P

*PEN* Penicillin susceptibility

*LVX* Levofloxacin susceptibility

*CAZ* Ceftazidime susceptibility

*CRO* Ceftriaxone susceptibility

*CLI* Clindamycin susceptibility

*AZM* Azithromycin susceptibility

(-) Indicates frozen isolate could not be successfully recovered.

*S* Sensitive, *I* Intermediate, *R*=Resistant

^a^ = CLSI breakpoints do not exist for ceftazidime. Results reported as Kirby-Bauer zone sizes.

^b^ Production of virulence factors reported as strains producing those factors; *P* positive (Strains produced this virulence factor), or *N*=negative

**Fig. 1:**
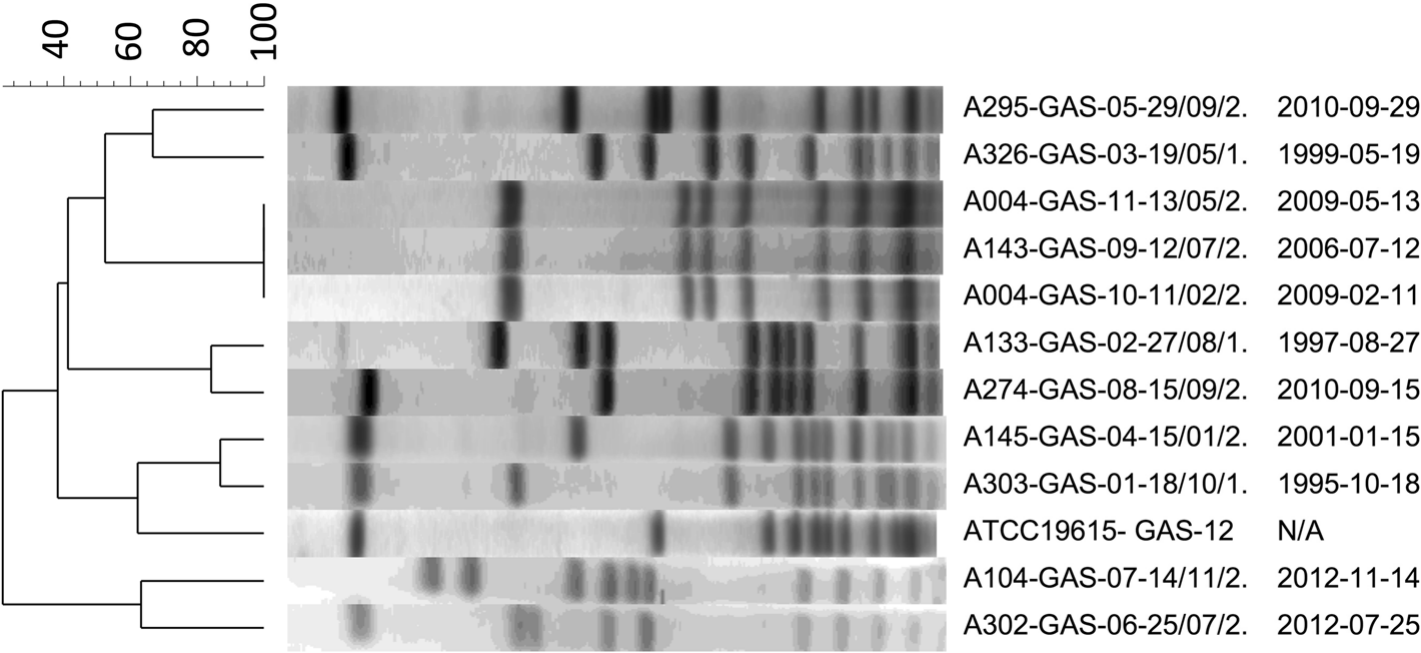
SmaI restriction digest pulse field gel electrophoresis of *S. pyogenes* recovered from CF sputum. A = patient number, date indicated as year/month/day

Genotypic assessment of strain relatedness to assess for natural history of infection and potential for patient-patient spread was evaluated. PFGE profiles of the 11 viable isolates from the biobank and a control strain ATCC 19615 were performed. Patient A004 isolated the same strain of GAS over three months demonstrating persistent carriage of a single isolate was possible. Two patients A004 and A143 had isolates with the same PFGE profile with both SmaI (Fig. 1) and ApaI (not shown), although these isolates were collected three years apart suggesting patient-patient transmission was not a factor.

## Discussion

Our study is the first to investigate the prevalence of GAS and its clinical effects in the adult CF population. *S. pyogenes* is a common organism that can affect healthy individuals of any age [14]. While GAS may transiently colonize the upper respiratory tract as a commensal organism (as is the case in 15 to 20 % of healthy children), it has also proven itself as a major human pathogen [24]. It is responsible for a broad spectrum of disease including pharyngitis, scarlet fever, rheumatic fever, cellulitis, necrotizing fasciitis, toxic shock syndrome, and pneumonia [14]. GAS infections can range from mild to life threatening; severe disease is not limited to those with chronic illness or immune compromise [14]. Globally, GAS is responsible for a significant burden of disease; every year it accounts for 110 million skin and soft tissue infections [11], 660 000 cases of invasive infection [11] and over 294 000 deaths [25].

From a pulmonary standpoint, GAS is an infrequent but important cause of pneumonia [26]. While it only accounts for a small percentage of community acquired pneumonia (CAP) in adults, *S. pyogenes* tends to cause more severe and invasive infection compared to common CAP pathogens [14]. Furthermore, GAS has a higher likelihood of resulting in necrotizing pneumonia, progression to empyema and/or hemorrhagic pleural effusion [13, 27]. Mortality rates for GAS pneumonia range between 20 and 38 %, which is similar to that of necrotizing fasciitis [13, 14].

Although *S. pyogenes* is a major global pathogen, little is known about its role in CF lung disease. Given the potential for GAS to cause exceptional virulence and the compromised innate immunity of CF lungs, we hypothesized that GAS may lead to adverse clinical outcomes in this population. There is a paucity of GAS epidemiologic data in the CF literature, with prior studies reporting prevalence of 0.8 % (2/258) [28] and 0.9 % (4/495) [29], respectively. Over 34 years, we identified that five percent of patients isolated GAS on at least one occasion. However, the presence of GAS increased the risk of PEx relative to the preceding clinic visit, particularly if present as the numerically dominant sputum pathogen. This finding may warrant treating individuals with GAS in their sputum with anti-GAS treatments in order to potentially avoid an ensuing PEx. However, other factors including exacerbation of chronically infecting pathogens, and inter-current upper respiratory viral illnesses could also have contributed (although these factors were just as likely in comparator clinical visits).

Within CF, it is clear that mere culture status may not convey the entire story. Indeed, differential pathogenic potential has been observed with the expression of a number of phenotypic traits of classical CF pathogens including *P. aeruginosa*, *Bcc*, and *S. aureus*. For example, compared to patients with chronic methicillin-sensitive *S. aureus* (MSSA) infection, those with chronic methicillin-resistant *S. aureus* (MRSA) have an increased risk of death [30]. Patients with MRSA have increased rates of lung function decline [31] and are less likely to recover lung function following PEx [32]. In patients with chronic *P. aeruginosa* infections, its conversion to a hyper-alginate producing, mucoid phenotype is associated with progressive decline in lung function, increased risk of hospitalization and reduced survival [8, 33–36]. The opposite appears true in *Bcc* chronically infected patients, where mucoidy appears protective and patients with non-mucoid isolates experience an exaggerated rate of clinical decline [37, 38]. Even the ability to persist within the CF lung seems to be influenced by specific phenotypic traits of *P. aeruginosa* causing initial infections [39]. The phenotypes that are associated with these strains may themselves not be directly involved in disproportionate lung disease, but rather they may be an indirect marker. Accordingly, we sought to characterize easily assayable and important virulence traits within infecting GAS strains to determine if these factors disproportionally modified PEx risk.

The GAS isolated from CF airways were typical of GAS reported in other diseases [40]. We identified variable expression of virulence factors, which has previously been reported [41]. Of the limited virulence factors assessed, expression did not increase risk for occurrence of PEx at the time of isolation. Almost all GAS isolates produced AI-2, a diffusible cell-cell signaling molecule enabling inter-species bacterial communication on cell density [42, 43]. Either directly (GAS mediated primary effects) or indirectly (through induction of quorum sensing in patients chronically colonized with pathogens such as *P. aeruginosa*), GAS may be able to trigger a PEx [44].

The antibiotic sensitivity profile of our strains was similar to that of other *S. pyogenes* epidemiologic studies [40, 45], with the exception of a complete absence of clindamycin resistance in our few isolates. Interestingly, our study along with several small studies [40, 45], have not found high rates of macrolide or fluoroquinolone resistance, as reported in larger studies (despite their frequent use in CF). Indeed, this may suggest that those GAS in CF are not unrecognized chronic endogenous lower respiratory tract flora in these individuals but rather newly acquired transient organisms not previously exposed to antibiotics. Importantly, our GAS strains were sensitive to antibiotics commonly used in the empiric treatment of CF PEx, although ceftazidime, an antibiotic commonly used in the empiric PEx management, should not be used where GAS is involved. Nearly all strains were sensitive to azithromycin; this raises the possibility that chronic azithromycin may suppress GAS and prevent initial colonization. Indeed, registry data suggests that 60–70 % of CF patients with chronic *P. aeruginosa* infection and 22 % of CF patients without chronic *P. aeruginosa* receive chronic macrolide therapy and this may account for its low observed incidence in our cohort [46, 47].

PFGE has been shown to be similarly effective at differentiating commonly infecting clones of GAS as other established typing modalities including *emm* gene typing [48–50]. Using PFGE we demonstrated strain persistence in those patients with repeated positive cultures, rather than repeated new infections with different strains. We did identify two patients with the same GAS isolate by PFGE, but propose this was unlikely to be patient-patient spread. Whereas typical CF pathogens are rare and opportunistic of the general population, GAS commonly colonizes the upper respiratory tract in the general population and common strains persist in locals for extended period of times [51, 52]. Furthermore, GAS from these patients were identified > 2.5 years apart with multiple negative cultures in the ensuing time period making CF patient-patient transmission biologically implausible.

Members of the genus *Streptococcus* have not traditionally been considered CF respiratory pathogens. However, using a combination of semi-selective agars and high density sampling, high rates of Viridans Group Streptococci (VGS) have universally been identified [53–55]. The *Streptococcus anginosus* group, in particular, is increasingly thought to have a role in CF and its emergence as numerically dominant organism has been observed in a large subset of PEx [54]. Traditional clinical microbiology protocols have been developed to overlook VGS. However, GAS is easily identifiable as beta-hemolysis is a defining feature (present in 99 % of isolates) [56], and as such is more likely to be distinguished from oropharyngeal streptococci using traditional culture techniques.

The role of bacteria in CF airways disease has been viewed through the lens of contributing to chronic progressive lung disease [57, 58]. Indeed, when assessing traditional CF pathogens such as *P. aeruginosa* and *B. cepacia* complex, as well as emerging organisms such as *S. maltophilia,* this model holds true. However, increasingly organisms not typically associated with CF airways disease including the Enterobactericeae, Pneumococcus, and GAS are seen to transiently colonize the airways [8, 59, 60]. The impact of these organisms on short-term and long-term outcomes are for the most part unknown. Given that transient colonization/infection with respiratory viruses has been shown to produce short term deleterious effects either through direct pathogenesis or indirectly through resident microflora, so too might transient bacterial pathogens [61]. Indeed, while an acute impact on patient well-being was observed with incident GAS infection, long term effects were not noted, nor should they be expected. This highlights the concept that emerging CF pathogens may not necessarily manifest as chronic infections, as is often seen with *P. aeruginosa, S. aureus,* and *Bcc*.

The main limitations of the study are the retrospective design and wide confidence intervals relating to small sample size and few events. Despite trying to account for factors that could influence the risk of PEx, we were unable to control for differences in chest physiotherapy quality and frequency as well as adherence to medications. We also lacked documentation regarding potential confounding viral and/or environmental triggers for PEx. Future studies assessing potential short-term impact of transient airway colonizers are warranted based on the data herein.

## Conclusion

GAS was an uncommon, transient lower respiratory pathogen in our population of adults with CF. In our cohort, identification of GAS in sputum, particularly as the dominant organism, was associated with an increased PEx risk. However, GAS colonization of the lower airways was transient and, as such, did not impact lung function and did not appreciably change the disease trajectory.

## Abbreviations

AB: Agrobacterium minimal medium
AI-2: Auto inducer 2
AZM: Azithromycin
BHI: Brain-heart infusion
CF: Cystic fibrosis
CO_2_: Carbon dioxide
CFU: Colony forming units
CAP: Community acquired pneumonia
CAZ: Ceftazidime
CBA: Columbia blood agar
CDA: Clindamycin
CLSI: Clinial and Laboratory Standards Institute
CRO: Ceftriaxone
E: Erythromycin
FEV_1_: Change in % predicted forced expiratory volume in one second
GAS: Group A Streptococcus
PEx: Pulmonary exacerbation
ICS: Inhaled corticosteroid
I: Intermediate
KB: Kirby Bauer
LABA: Long acting beta agonist
LEV: Levofloxacin
MHBA: Mueller-Hinton blood agar
MSSA: Methicillin sensitive *Staphylococcus aureus*
OD: Optical denstity
P: Penicillin G
PFGE: Pulse field gel electrophoresis
R: Resistant
S: Susceptible
TSY: Trypticase soy yeast agar
UPGMA: Unweighted pair-group method with arithmetic mean
